# Public Mental Health: Kernstück oder Stiefkind von Public Health?

**DOI:** 10.1007/s00103-023-03670-y

**Published:** 2023-03-03

**Authors:** Steffi G. Riedel-Heller, Ulrich Reininghaus, Georg Schomerus

**Affiliations:** 1 Institut für Sozialmedizin, Arbeitsmedizin und Public Health (ISAP), Medizinische Fakultät, Universität Leipzig, Philipp-Rosenthal-Str. 55, 04103 Leipzig, Deutschland; 2 Abteilung Public Mental Health, Zentralinstitut für Seelische Gesundheit, Medizinische Fakultät Mannheim, Universität Heidelberg, Mannheim, Deutschland; 3 Centre for Epidemiology and Public Health, Health Service and Population Research Department, Institute of Psychiatry, Psychology & Neuroscience, King’s College London, London, Großbritannien; 4 ESRC Centre for Society and Mental Health, King’s College London, London, Großbritannien; 5 Klinik und Poliklinik für Psychiatrie und Psychotherapie, Universität Leipzig, Leipzig, Deutschland

**Keywords:** Public Mental Health, Public Health, Psychische Erkrankungen, Bevölkerung, Prävention, Public mental health, Public health, Mental disorders, Population, Prevention

## Abstract

Der vorliegende Beitrag gibt eine Übersicht zum Selbstverständnis, zu Forschungs- und Handlungsfeldern von Public Mental Health. Es wird deutlich, dass die psychische Gesundheit ein zentrales Element der Bevölkerungsgesundheit ist und eine einschlägige Wissensbasis zu diesem Themenfeld existiert. Darüber hinaus werden Entwicklungslinien des an Bedeutung gewinnenden Bereiches in Deutschland aufgezeigt. Obgleich es im Feld von Public Mental Health wichtige aktuelle Initiativen wie die Etablierung einer Mental Health Surveillance (MHS) und die Offensive Psychische Gesundheit gibt, entspricht die Positionierung im Feld nicht der bevölkerungsmedizinischen Relevanz psychischer Erkrankungen.

## Abstract (en)

This article provides an overview of the self-conception, research, and fields of action of public mental health. It is becoming clear that mental health is a central element of public health and that a relevant knowledge base exists on this topic. In addition, lines of development of this field, which is gaining in importance in Germany, are shown. Although there are important current initiatives in the field of public mental health, such as the establishment of a Mental Health Surveillance (MHS) and the Mental Health Offensive, the positioning in the field does not correspond to the relevance of mental illness in population medicine.

## Psychische Gesundheit – zentrale Rolle für die Bevölkerungsgesundheit

Psychische Erkrankungen sind häufig und folgenschwer. Zahlen des Robert Koch-Institutes (RKI) zeigen, dass ungefähr jeder Dritte in einem Zeitraum von 12 Monaten eine oder mehrere psychische Erkrankungen aufweist [1]. Das entspricht rund 17,8 Mio. betroffenen Personen, von denen pro Jahr nur 18,9 % Kontakt zu Leistungsanbietern aufnehmen [2]. Am häufigsten sind Angsterkrankungen, gefolgt von depressiven Erkrankungen und Erkrankungen in Zusammenhang mit Substanzabhängigkeit oder -missbrauch [3]. Bei alten und hochaltrigen Menschen sind Demenzerkrankungen häufig [4]. Die Konsequenzen psychischer Erkrankungen für den Einzelnen, sein soziales Umfeld, aber auch für die Solidargemeinschaft sind schwerwiegend.

Die Gesundheitsberichte verschiedener gesetzlicher Krankenkassen in Deutschland verzeichnen einen kontinuierlichen Anstieg an Arbeitsunfähigkeitstagen. Von 2000 bis 2019 gab es bei den Fehltagen aufgrund von psychischen Erkrankungen insgesamt einen Anstieg um 137 % [5]. Im Jahr 2020 dauerte dabei eine durchschnittliche Krankschreibung aufgrund einer psychischen Erkrankung (ICD-10 F00–F99) der DAK-Versicherten in Deutschland 38,8 Tage und ist damit in den letzten Jahrzehnten kontinuierlich gestiegen und deutlich länger als bei anderen Erkrankungen. Psychische Erkrankungen sind heute mit 42 % die häufigste Ursache für krankheitsbedingte Frühberentungen [6]. Die Berentungen erfolgen zudem früher als bei anderen Erkrankungen, im Durchschnitt mit 48 Jahren [7]. Die Global Burden of Disease Study [8] ist der umfassendste Ansatz, Lebenserwartungen, Krankheitslasten und Risikofaktoren weltweit zu schätzen. Psychische Erkrankungen beginnen oft frühzeitig und haben Konsequenzen für den gesamten Lebenslauf. So werden in Westeuropa bei den 20- bis 24-Jährigen bis zu 40 % der individuellen Krankheitslasten, sogenannte „years lived with disability“ (YLDs), von psychischen Erkrankungen verursacht. Über alle Altersgruppen hinweg sind es 21 %. Die Krankheitslast, die mit psychischen Erkrankungen verbunden ist, betrifft insbesondere vulnerable Gruppen, wie z. B. Kinder in prekären sozioökonomischen Situationen, traumatisierte Menschen, Obdachlose oder Menschen, die eine psychische und somatische Komorbidität haben. Psychische Erkrankungen gehören zudem zu den kostenintensivsten Erkrankungen. Die Gesamtkosten aufgrund psychischer Erkrankungen werden für Deutschland auf rund 147 Mrd. € pro Jahr geschätzt, was einem Anteil von 4,8 % am Bruttoinlandsprodukt entspricht [9]. Diese Fakten verdeutlichen die Relevanz von psychischen Erkrankungen auf Bevölkerungsebene.

## Womit beschäftigt sich Public Health?

Seit Jahrhunderten sind Public-Health-Aspekte Teil der klinischen Medizin. Es waren Ärzte, die für bessere sanitäre Bedingungen kämpften oder körperliche Aktivität propagierten. Das Lehrbuch von David Klemperer definiert Public Health als „die Wissenschaft und Praxis zur Vermeidung von Krankheiten, zur Verlängerung des Lebens und zur Förderung von physischer und psychischer Gesundheit unter Berücksichtigung einer gerechten Verteilung und einer effizienten Nutzung der vorhandenen Ressourcen“ [10]. Public-Health-Maßnahmen zielen primär auf die Gesunderhaltung der Bevölkerung und einzelner Bevölkerungsgruppen. Damit wird die Perspektive der klinischen Medizin, die sich in erster Linie auf Individuen und ihre Erkrankungen richtet, erweitert. Forschungs- und Handlungsfelder umfassen die Epidemiologie, die Prävention und Gesundheitsförderung, die Versorgungsforschung, die Gesundheitssystemforschung, die Gesundheitsökonomie, Politikberatung und Monitoring der Entscheidungen in verschiedenen Politikfeldern („health in all policies“) als auch Aspekte der Patienten- und Bürgerbeteiligung.

Deutschland war im 19. und im frühen 20. Jahrhundert das Pionierland bei der Entwicklung einer wissenschaftlich begründeten öffentlichen Gesundheitsfürsorge. Bestimmte Strömungen innerhalb der öffentlichen Gesundheitsfürsorge, in denen die Gesundheit eines „Volkskörpers“ auf Kosten diskriminierter Minderheiten verbessert werden sollte („Rassenhygiene“, „Eugenik“, „Euthanasie“) wurden im Nationalsozialismus aufgegriffen, erweitert und mit der systematischen Ermordung von 216.000 Menschen mit körperlichen, geistigen und seelischen Behinderungen während der Zeit des Nationalsozialismus grausam auf die Spitze getrieben [11]. Bevölkerungsbezogene Sichtweisen innerhalb der Medizin waren deshalb nach dem Krieg in Deutschland diskreditiert. Dies führte zu einem Bedeutungsverlust der öffentlichen Gesundheit in den Nachkriegsjahrzehnten und zu einem substanziellen Rückstand im internationalen Vergleich [12]. Diese Einbrüche sind bis zum heutigen Tag nicht vollständig überwunden. Erst Ende der 1980er-Jahre ist es zu einer Wiederbelebung von Public Health in Deutschland gekommen, wobei traditionell Aspekte der körperlichen Gesundheit ganz im Mittelpunkt standen. Es entstanden zunehmend Studiengänge an Universitäten und Fachhochschulen; aktuell sind es 21 Studiengänge an 19 Hochschulen (10 an Universitäten und 9 an Fachhochschulen [13]). Eine Stärkung von Public Health wird als entscheidender Ansatz zur Schaffung gesundheitsförderlicher Lebensbedingungen und mehr gesundheitlicher Chancengleichheit gesehen [14].

Public Health hat viele Akteure. Ein wesentlicher Praxis-Akteur ist der Öffentliche Gesundheitsdienst (ÖGD). Der ÖGD stellt neben der ambulanten und stationären Versorgung jedoch eine sehr schmale dritte Säule der Gesundheitsversorgung dar. Im Hinblick auf die psychische Gesundheit haben die Sozialpsychiatrischen Dienste des ÖGD eine wichtige Rolle (z. B. Zugang zur Regelversorgung, Prävention) inne. Die COVID-19-Pandemie hat die langjährigen strukturellen Defizite im Öffentlichen Gesundheitsdienst offengelegt und mit dem Pakt für den ÖGD den Beginn einer längst überfälligen Stärkung markiert [15].

## Was ist Public Mental Health?

Wenn psychische Gesundheit und psychische Erkrankungen im Mittelpunkt der Public-Health-Forschung oder -Praxis stehen, sprechen wir auch von Public Mental Health. Public Mental Health befasst sich dementsprechend mit der Prävention und Identifikation von psychischen Erkrankungen in der Bevölkerung und mit deren Management, aber auch mit der Evaluation der Behandlung und der Förderung bzw. dem Schutz der psychischen Gesundheit. Public Mental Health geht damit in seinen Aktivitäten über das traditionelle medizinische Setting hinaus und umfasst Aktivitäten in Schulen, Betrieben, Nachbarschaften, Gemeinden und Ländern. Public Mental Health umfasst alle in Tab. 1 genannten Forschungs- und Handlungsfelder in Bezug auf psychische Erkrankungen. Konzeptionelle Überschneidungen zu gemeinde- und sozialpsychiatrischen Inhalten werden deutlich.

**Table 1:** 

Forschungsfelder	Handlungsfelder
Epidemiologie	Erfassung der Häufigkeit und Verteilung von psychischen Erkrankungen in der Gesellschaft, einschließlich der sozial bedingten gesundheitlichen Ungleichheit Untersuchung der Ursachen von psychischer Gesundheit und psychischer Krankheit, insbesondere sozialer und umweltbedingter Einflussfaktoren auf die psychische Gesundheit
Prävention und Gesundheitsförderung	Untersuchung von Wirksamkeit und Effizienz unterschiedlicher Maßnahmen zur Förderung der psychischen Gesundheit und Prävention psychischer Erkrankungen sowie deren Implementierung
Versorgungsforschung	Erfassung des aktuellen und prospektiven Bedarfes an präventiver, kurativer, rehabilitativer, pflegerischer Versorgung der Bevölkerung in Bezug auf psychische Erkrankungen Untersuchung der Wirksamkeit und Effizienz unterschiedlicher Maßnahmen zur Behandlung psychischer Erkrankungen, Prüfung und Fortentwicklung bestehender Strukturen und Prozesse im Sinne einer effektiven und effizienten gesundheitlichen Versorgung psychisch Kranker und Förderung der psychischen Gesundheit
Gesundheitssystemforschung	Entwicklung eines Gesundheitssystems, das allen Bürgern eine hohe Versorgungsqualität und einen gleichberechtigten, ihrem individuellen Bedarf entsprechenden und bezahlbaren Zugang zu entsprechenden Versorgungsleistungen in Bezug auf psychische Erkrankungen bietet
Gesundheitsökonomie	Steuerung und Finanzierung der Versorgung im Gesundheitswesen in Bezug auf psychische Erkrankungen
Politik	Politikberatung und Monitoring von Entscheidungen in diversen Politikfeldern hinsichtlich ihrer Effekte für die psychische Gesundheit der Bevölkerung („mental health in all policies“; „equity in all policies“), Politikberatung in Bezug auf die Versorgung psychisch Erkrankter
Patienten- und Bürgerbeteiligung	Beteiligung von Patienten und ihren Angehörigen sowie Bürgerinnen und Bürgern an der Gestaltung des Gesundheitswesens und an gesundheitsrelevanten Entscheidungen in allen Politikfeldern, Vorreiterrolle der Psychiatrie in Bezug auf Trialog und Betroffenenbeteiligung für andere Bereiche der Medizin

Obgleich Public Mental Health international eine wachsende Disziplin ist, wurde sie von der Mutterdisziplin für lange Zeit ignoriert und gilt noch immer als unterschätzt [16]. Dafür gibt es vor allem historische Gründe. Die traditionelle Public-Health-Forschung fokussierte in ihren Anfängen auf Infektionserkrankungen und Hygieneaspekte und hatte damit einen ganz anderen Schwerpunkt. Zudem wurde die Psychiatrie lange als ein Fach wahrgenommen, das mit seinem phänomenologischen Zugang auf der dyadischen persönlichen Beziehung der Behandler mit seinen Patienten fußt und zudem besondere Herausforderungen in der Falldefinition und Operationalisierung aufweist [17]. In Deutschland wurde Public Mental Health diskreditiert durch die schwere Hypothek aus der Zeit des Nationalsozialismus, in der aus der damaligen unheilvollen Verbindung von Bevölkerungsmedizin und Psychiatrie enormes Leid für psychisch kranke Menschen und ihre Angehörigen erwuchs. Die genannten Hürden erschwerten einen bevölkerungsmedizinischen Zugang zur psychischen Gesundheit, besonders in Deutschland. Zudem waren Informationen zur Häufigkeit und den Folgen psychischer Erkrankungen und zu effektiven präventiven Interventionen und Behandlungsmöglichkeiten wie auch zum Stigma von psychischen Erkrankungen in der Bevölkerung historisch gesehen rar [18]. Das hat sich grundsätzlich geändert. Heute liegen solide Wissensbestände zu den Forschungs- und Handlungsfeldern von Public Health in Bezug auf psychische Erkrankungen vor. Eine vereinfachte Zusammenfassung findet sich in der Infobox.

Vertiefend soll an dieser Stelle nur auf Befunde zur Gesundheitsförderung und Prävention psychischer Erkrankungen eingegangen werden. Beide Begriffe stellen unterschiedliche Blickwinkel auf dasselbe Ziel dar, nämlich Erkrankungen vorzubeugen. Prävention betont dabei die Reduktion von Risikoverhalten und Risikofaktoren in Person und Umwelt; Gesundheitsförderung hebt auf die Stärkung von Ressourcen und gesundheitsunterstützenden Umwelten ab [20]. Es existiert eine umfassende internationale Literatur zur Effektivität von Präventionsprogrammen im Bereich der psychischen Gesundheit bei Kindern, Jugendlichen, Erwachsenen und alten Menschen [20–22]. Gleiches gilt auch für Transitionsphasen wie die Schwangerschaft, die Umstellung auf Elternschaft als auch für Trauer und Verlust im Alter [23]. Dabei eröffnet sich ein enormes Handlungsfeld mit den verschiedensten verhaltens- und verhältnispräventiven Ansätzen in verschiedenen Settings.

Beispielhaft wird hier nur die Prävention von Depression, Psychosen und kognitiven Erkrankungen angesprochen. Weitere Bereiche wie die Suizidprävention haben große Bedeutung in Public Mental Health. Zu den Maßnahmen der *Depressionsprävention* zählen unter anderem Lebensstilinterventionen (körperliche Aktivität, Bewegungsförderung, Schlafhygiene), soziale Interventionen (Förderung sozialer Aktivitäten, Stabilisierung sozialer Beziehungen, soziale Unterstützung) sowie psychologische Interventionen (Stressmanagement, Förderung von positiven Aktivitäten und Resilienz, Erhöhung der Entspannungs- und Genussfähigkeit [24, 25]). Zudem weiß man mehr darüber, welche Zielgruppen von spezifischen präventiven Maßnahmen profitieren. In einer klassischen Übersicht, in die mehr als 30 randomisierte kontrollierte Studien (RCTs) eingeschlossen wurden, konnten Muñoz et al. zeigen, dass sich Neuerkrankungen einer Depression (Major Depression) um 20 % bis zu 50 % reduzieren lassen [26]. In den letzten Jahren haben sich die Befunde zur Wirksamkeit von Bewegungsintervention [27] und zu onlinebasierten psychologischen oder psychoedukativen Interventionen gemehrt [28]. Dies ist aus Public-Mental-Health-Perspektive besonders interessant, da diese Interventionen und Onlinetools skalierbar sind und große Bevölkerungsgruppen erreicht werden können. Intensiv beforscht ist auch das Feld der *Psychoseprävention*. Im Rahmen von RCTs in Hochrisikogruppen konnte gezeigt werden, dass diese Interventionen ganz überwiegend mit einem günstigeren Symptomverlauf, einer besseren sozialen Anpassung und weniger Übergängen in die Psychose verbunden waren [29–31].

Der *Prävention kognitiver Erkrankungen* im Alter wird gegenwärtig besondere Aufmerksamkeit geschenkt [32]. Es werden Brain-Health-Agenden^1^ gefordert und es gibt Empfehlungen der Weltgesundheitsorganisation (WHO) zur Risikoreduktion [33]. Entsprechend den Herausforderungen bedingt durch die demografische Entwicklung wurden große, bevölkerungsbezogene multimodale und randomisierte Präventionsstudien in Europa auf den Weg gebracht [34]. Diese zielen auf die Verbesserung der kognitiven Funktion bzw. auf die Verzögerung von Demenzeintritt und Behinderung. Für die erste deutsche Studie dieser Art (AgeWell.de) werden bald Ergebnisse erwartet [35]. Zu den zentralen Komponenten dieser multimodalen Interventionen im Rahmen der Demenzprävention zählen Ernährungsberatung, Bewegungsförderung, kognitives Training, die Stimulierung sozialer und kognitiver Aktivität sowie Monitoring und Management von metabolischen und vaskulären Risikofaktoren. Viele der beispielhaft genannten Interventionen sind verhaltenspräventiv. Verhältnispräventive Ansätze werden zunehmend ausgelotet.

## Public Mental Health – Herzstück oder Stiefkind von Public Health?

Entsprechend den gängigen Kriterien, die Gesundheitswissenschaftler nutzen, um zu entscheiden, welche Gesundheitsprobleme eine Priorität in Public Health haben, wie z. B. Umfang und Folgenschwere des Problems, Verfügbarkeit von Interventionen, ökonomischer und sozialer Impact [36], sollten psychische Erkrankungen eigentlich zum Public-Health-Problem Nummer eins avancieren.

Vor 10 Jahren machte der Chief Medical Officer’s Report 2013 im Vereinigten Königreich erstmals unter dem Titel „Public Mental Health Priorities: Investing in the Evidence“ die psychische Gesundheit der Bevölkerung zum Topthema [37]. In der aktuellen Strategie von Public Health England ist Mental Health noch immer als Priorität explizit gelistet [38]. Das ist in Deutschland nicht ganz so. Gleichwohl gab und gibt es einschlägige Forschungsarbeiten, die den Grenz- und Spannungsbereich von Psychiatrie und Gesellschaft in den Blick nehmen. Sie firmieren jedoch oft unter anderen Begriffen, z. B. als psychiatrische Epidemiologie, sozialpsychiatrische Forschung, psychiatrische Einstellungsforschung oder Versorgungsforschung. Zum Oberbegriff Public Mental Health wird erst in den letzten Dekaden Referenz genommen. Holzinger et al. analysierten vor knapp 20 Jahren die Forschungsaktivitäten zu Public Mental Health im deutschsprachigen Raum, die sich damals auf wenige psychiatrische Universitätskliniken, das Max-Planck-Institut für Psychiatrie (MPI) in München, das Zentralinstitut für Seelische Gesundheit (ZI) in Mannheim und wenige sozialmedizinische Institute konzentrierten [39]. Die Forschungslandschaft hat sich seitdem international, aber auch in Deutschland deutlich verbreitert.

Aktuell gibt es in Deutschland Initiativen, die zeigen, dass dem Bereich Public Mental Health mehr Bedeutung zugemessen wird. 3 Beispiele sollen dies verdeutlichen: (1) die Verankerung der psychischen Gesundheit in den Gesundheitszielen, (2) die Initiierung einer Mental Heath Surveillance (MHS) und (3) die „Offensive Psychische Gesundheit“:

- Die Relevanz psychischer Erkrankungen wird – zumindest in gewissem Maße – in den (1) *nationalen Gesundheitszielen* widergespiegelt: Die für die psychische Gesundheit der Bevölkerung relevanten Gesundheitsziele, „depressive Erkrankungen verhindern, früh erkennen und nachhaltig behandeln“ sowie „Gesund älter werden“, „Reduktion des Tabakkonsums“ und „Alkoholkonsum reduzieren“, werden als Kernbereiche präventiver Maßnahmen explizit benannt, womit der Depressions‑, Demenz- und Suchtprävention ein wichtiger Stellenwert beigemessen wird. Darüber hinaus gibt es Anknüpfungspunkte an das Gesundheitsziel, „gesund aufwachsen“, im Hinblick auf besondere Maßnahmen für Kinder psychisch erkrankter Eltern und die Psychoseprävention bei Adoleszenten [40]. Das Robert Koch-Institut, das deutsche Public-Health-Institut, hat seine Expertisen zur psychischen Gesundheit in einem eigenen Fachbereich (Fachgruppe 26) gebündelt, der nun in die Abteilung für Epidemiologie und Gesundheitsberichterstattung eingebettet ist [41].
- Ein wichtiges Projekt dieser Fachgruppe stellt die Etablierung einer (2) *Mental Health Surveillance (MHS)* in Deutschland dar, das 2019 startete. Dabei wurde ein Set handlungsleitender Kernindikatoren für die psychische Gesundheit der Bevölkerung definiert [42, 43]. Diese sollen nun systematisch und kontinuierlich mittels verfügbarer Daten quantifiziert werden. Auf dieser Grundlage können regelmäßige Aussagen zur psychischen Gesundheit und zu deren Entwicklung über die Zeit in Deutschland getroffen werden. Obgleich diese Informationen von zentraler Bedeutung sind, sind die Finanzierung und Weiterführung dieses wichtigen Projektes über 2023 hinaus noch offen.
- Von 2020 bis 2021 wurde die (3) *Offensive Psychische Gesundheit* vom Bundesministerium für Arbeit und Soziales (BMAS), dem Bundesministerium für Gesundheit (BMG) und dem Bundesministerium für Familie, Senioren, Frauen und Jugend (BMFSFJ) sowie zentralen Akteuren aus dem Bereich der Prävention ins Leben gerufen. Die beteiligten Bundesministerien nehmen dabei wichtige präventionsrelevante Lebenswelten wie Arbeit, Schule und Familie in den Blick [44]. Mit der Offensive Psychische Gesundheit soll eine gesellschaftliche Debatte angestoßen werden, um offener über psychische Belastungen zu sprechen. Zudem werden Präventionsangebote stärker miteinander vernetzt.

Diese 3 Bespiele zeigen, dass Public Mental Health in Deutschland in den letzten Jahren in der Wahrnehmung an Bedeutung gewonnen hat. Gleichwohl nimmt Public Mental Health nicht den Platz ein, der dem Feld aufgrund der bevölkerungsmedizinischen Bedeutung gebührt. Das hat sich besonders in der COVID-19-Pandemie gezeigt. So standen in der initialen Pandemie nahezu ausschließlich virologische und infektionsepidemiologische Aspekte im Vordergrund und erst schrittweise wurden die psychischen Folgen der Pandemie erkannt. Heute weiß man, dass psychische Reaktionen im Hinblick auf Ängstlichkeit, Depressivität und Belastungserleben insbesondere bei Jugendlichen und jungen Erwachsenen besonders ausgeprägt waren. Junge Menschen haben die Maßnahmen des Gesundheitsschutzes (z. B. Lockdown) besonders getroffen [45]. Alte Menschen waren initial überraschend psychisch stabil [46, 47]. Man geht davon aus, dass psychische Reaktionen auf sehr belastende Erlebnisse in einem gewissen Rahmen normal und nicht zwingend Ausdruck einer längerfristigen psychischen Erkrankung sind. Gleichwohl sind diese kurzfristigen Pandemiefolgen für die Psyche relevant. Hinsichtlich der langfristigen Folgen ist von einer rezessionsbedingten Zunahme psychischer Erkrankungen auszugehen. Die Zusammenhänge zwischen Wirtschaftskrisen und häufigerem Auftreten psychischer Erkrankungen sind lange bekannt [48]. Hahad et al. stellen einen anderen Mechanismus in den Mittelpunkt: Sie zeigen auf der Grundlage von Ebola- und Zika-Virus-Ausbrüchen, dass maladaptive Verhaltensweisen durch erhöhte psychische Belastungen und Ängste die Implementierung von Public-Health-Maßnahmen beeinträchtigen und letztlich über Angst- und Verdrängungsspiralen zu einer stärkeren Ausbreitung beitragen können [49]. Die Förderung und Erhaltung der psychischen Gesundheit sollte ein zentrales Element im Pandemiemanagement sein – und zwar von Beginn an. Zeitnahe Informationen zur psychischen Gesundheit, z. B. im Rahmen einer Mental Health Surveillance, sind dabei unerlässlich. Aktuelle Zahlen des RKI bis Juni 2022 geben hinsichtlich der Belastung der Bevölkerung keine Entwarnung [50].

## Public Mental Health – wo geht die Reise hin?

Psychische Erkrankungen spielen schon jetzt eine zentrale Rolle für die Bevölkerungsgesundheit. Aktuelle große gesellschaftliche Entwicklungslinien, auch Megatrends genannt, deuten auf wachsende Herausforderungen für die psychische Gesundheit der Bevölkerung hin. 4 dieser großen Entwicklungstrends und ihre Auswirkungen sollen kurz erläutert werden:

- Bei der zunehmenden *Individualisierung* geht es um die Herauslösung aus historisch vorgegebenen Sozialformen und Bindungen und damit auch um den Verlust von traditionellen Sicherheiten. Idealerweise ist dies mit einer neuen Art von sozialer Einbindung verbunden, was nicht allen Menschen gelingt [51]. Schon vor der Pandemie waren in Deutschland 12,3 % der 18- bis 79-Jährigen einsam, bei den über 80-Jährigen ist es ein Drittel [52]. Mit der Pandemie nahm die Einsamkeit zu [53]. Wir wissen, dass Einsamkeit Auswirkungen auf die Gesundheit hat – die größten Auswirkungen wurden jedoch auf die psychische Gesundheit und das allgemeine Wohlbefinden beobachtet [54].
- Ein weiterer Megatrend ist die *Urbanisierung*. In Europa leben 74,5 % der Menschen in Städten, ihr Anteil ist wachsend. Auch diese Entwicklung wird zur Herausforderung, denn wir wissen zum Beispiel, dass ein höherer Anteil von Grünflächen in der Wohnumgebung mit einem niedrigeren Risiko für bestimmte psychische Erkrankungen verbunden ist [55].
- Mit der *demografischen Entwicklung* wächst der Anteil alter und besonders hochaltriger Menschen. Damit werden auch Demenzerkrankungen zur zentralen Herausforderung von sogenannten Silver Societies^2^.
- Zudem wirkt sich die *Klimakrise* negativ auf die psychische Gesundheit der Menschen aus und wird ein wichtiges zukünftiges Forschungs- und Aktionsfeld von Public Mental Health werden [56].

Es wird deutlich, dass Public Mental Health zentrale Zukunftsthemen bearbeitet und auch in Deutschland ein prosperierendes Feld ist. Es kann nicht mehr als Stiefkind von Public Health bezeichnet werden.

Gleichwohl ist erheblicher Entwicklungsbedarf auszumachen. Es bleibt eine Herausforderung, dieses Fach in seiner Interdisziplinarität abzubilden und den Diskurs im Spannungsfeld von Public Health, psychiatrischer Epidemiologie, psychischer Gesundheitsförderung, Prävention und Versorgungsforschung zu führen, um sein innovatives Potenzial vollständig auszuschöpfen [57]. In den einschlägigen wissenschaftlichen Fachgesellschaften wie der Deutschen Gesellschaft für Psychiatrie und Psychotherapie, Psychosomatik und Nervenheilkunde (DGPPN), der Deutschen Gesellschaft für Sozialmedizin und Prävention (DGSMP) und der Deutschen Gesellschaft für Public Health (DGPH) haben sich Referate und Fachgruppen zu diesem Thema gegründet, die fachgesellschaftsübergreifend in engem Austausch agieren. Und last, but not least – die Entwicklung von Public Mental Health wird in die gesamte weitere Entwicklung des Public-Health-Systems in Deutschland eingebettet sein. Dafür liegen strategische Vorschläge auf dem Tisch, die zwischen 2017 und 2021 auf Initiative des Zukunftsforums Public Health (ZfPH) erarbeitet wurden [14]. Eine Mental Health Surveillance (MHS), die eine aktuelle Datengrundlage zur psychischen Gesundheit der deutschen Bevölkerung liefert, ist in diesem Kontext eine wesentliche Grundlage für weitere gezielte Schritte.

### Infobox Public Mental Health – Grundlegende Wissensbestände [19]

Psychische Erkrankungen sind häufig und mit einer hohen Krankheitslast verbunden.

Psychische Erkrankungen entwickeln sich in der Interaktion von biologischen, psychologischen und sozialen Faktoren. Obgleich die sozialen Faktoren ein zentrales Element für die Verbesserung der psychischen Gesundheit sind, bedürfen diese der weiteren Auslotung, insbesondere vor dem Hintergrund tiefgreifender sozialer Wandlungsprozesse in der Gesellschaft.

Psychische und körperliche Gesundheit sind untrennbar verbunden. Die Gesundheit insgesamt wird von einer verbesserten psychischen Gesundheit profitieren, insbesondere von Public-Mental-Health-Interventionen.

Die Prävention psychischer Erkrankungen ist möglich. Studien zur Gesundheitsförderung und Prävention psychischer Erkrankungen zeigen ermutigende Resultate, wobei die Datenbasis ausbaufähig ist.

Fortentwicklungen psychiatrischer Versorgungssysteme haben das Potenzial, die Situation psychisch Kranker zu verbessern. Das Stigma psychischer Krankheit verstärkt die Belastung der Betroffenen über die ursprünglichen Krankheitssymptome hinaus. Es beeinflusst den Verlauf von psychischen Krankheiten negativ und stellt eine wichtige Hürde bei der Behandlung dar. Die Entstigmatisierung ist ein wichtiges Ziel von Public-Mental-Health-Interventionen.

## Footnotes

1 Mit Brain Health bezeichnet die WHO ein Konzept, das zunehmend nicht nur im Gesundheitsbereich, sondern in der Gesellschaft insgesamt diskutiert wird. Brain Health wird dabei definiert als der Zustand der Gehirnfunktion in allen kognitiven, sensorischen, sozial-emotionalen, verhaltensmäßigen und motorischen Bereichen, der es dem Menschen ermöglicht, sein volles Potenzial im Laufe seines Lebens auszuschöpfen.

2 Silver Society: eine Gesellschaft, in der ein substantieller Teil der Menschen älter als 60 Jahre ist.
